# Metabolomic profile differs between LADA and type 1 diabetes identifying tryptophan metabolism as a pathway involved in the heterogeneity of autoimmune diabetes

**DOI:** 10.1007/s00125-026-06777-4

**Published:** 2026-06-26

**Authors:** Ernesto Maddaloni, Samantha Pezzica, Rocco Amendolara, Silvia Sabatini, Gabriele Mocciaro, Guido Sebastiani, Luca D’Onofrio, Fabrizia Carli, Marta Tesi, Mara Suleiman, Luca Navarini, Laura Nigi, Caterina Formichi, Marta Vomero, Marco Minerba, Piero Marchetti, Francesco Dotta, Raffaella Buzzetti, Amalia Gastaldelli

**Affiliations:** 1 Department of Experimental Medicine, Sapienza University, Rome, Italy; 2 Cardiometabolic Risk Unit, Institute of Clinical Physiology-National Research Council, Pisa, Italy; 3 Diabetes Research Unit, Department of Medicine, Surgery and Neurosciences, University of Siena, Siena, Italy; 4 Fondazione Umberto Di Mario ONLUS, Toscana Life Science, Siena, Italy; 5 Department of Clinical and Experimental Medicine, University of Pisa, Pisa, Italy; 6 Clinical and Research Section of Rheumatology and Clinical Immunology, Fondazione Policlinico Universitario Campus Bio-Medico, Rome, Italy; 7 Rheumatology and Clinical Immunology, Department of Medicine, University of Rome Campus Bio-Medico School of Medicine, Rome, Italy; 8 Tuscany Centre for Precision Medicine (CReMeP), Siena, Italy

**Keywords:** Cytokines, Islets, Kynurenine, LADA, Latent autoimmune diabetes in adults, Lipids, Metabolomics, Rheumatoid arthritis, Tryptophan, Type 1 diabetes

## Abstract

**Aims/hypothesis:**

The heterogeneity of autoimmune diabetes may be associated with variable metabolic alterations. Our aim was to investigate differences in the metabolomic and lipidomic profile of autoimmune diseases and to identify pathways linked to beta cell damage. To this end, we compared latent autoimmune diabetes in adults (LADA) and type 1 diabetes, also comparing them with rheumatoid arthritis (RA), a related autoimmune condition, and healthy control participants.

**Methods:**

Metabolomic and lipidomic analyses were performed for 136 individuals (49 with LADA, 44 with type 1 diabetes, 29 with RA and 14 control participants). Omics of pancreatic islets from healthy donors were also evaluated after in vitro treatment with proinflammatory cytokines.

**Results:**

LADA and type 1 diabetes differed from RA in terms of lipidomics and metabolomics. Phosphatidylethanolamines, ceramides, lysophosphatidylcholine and several metabolites at the entry sites of the tricarboxylic acid cycle were higher in type 1 diabetes compared with LADA. In pancreatic islets treated with proinflammatory cytokines, tryptophan concentration was reduced by 80%, indicating the activation of tryptophan metabolism in response to the inflammatory stimulus. In people with autoimmune disorders, kynurenine/tryptophan ratio (Kyn/Trp), a marker of tryptophan pathway activation, was higher than in the control group (Kyn/Trp ratio in control group, median [25th–75th percentile]: 0.014 [0.012–0.019]), with a progressive decline from RA (0.027 [0.022–0.032]) to LADA (0.021 [0.018–0.024]) and then to type 1 diabetes (0.018 [0.014–0.022]), ANCOVA *p*<0.0001. In LADA, Kyn/Trp was directly associated with fasting C-peptide levels in the multivariate regression model accounting for confounders (*p*=0.038).

**Conclusions/interpretation:**

Metabolomic and lipidomic profiles differ between LADA and type 1 diabetes and vs RA, providing new insights into the heterogeneity of autoimmune diabetes. Our results confirm the involvement of tryptophan metabolism in autoimmune disorders, suggesting that the impaired activation of this pathway of immune tolerance in LADA is less pronounced than in type 1 diabetes, consistent with its milder degree of beta cell loss.

**Graphical Abstract:**

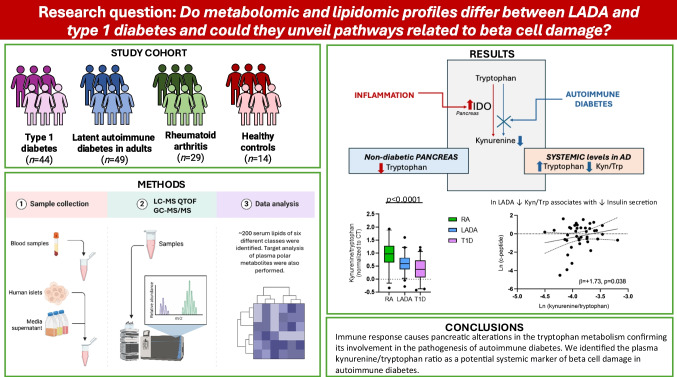

**Supplementary Information:**

The online version contains peer-reviewed but unedited supplementary material available at 10.1007/s00125-026-06777-4.


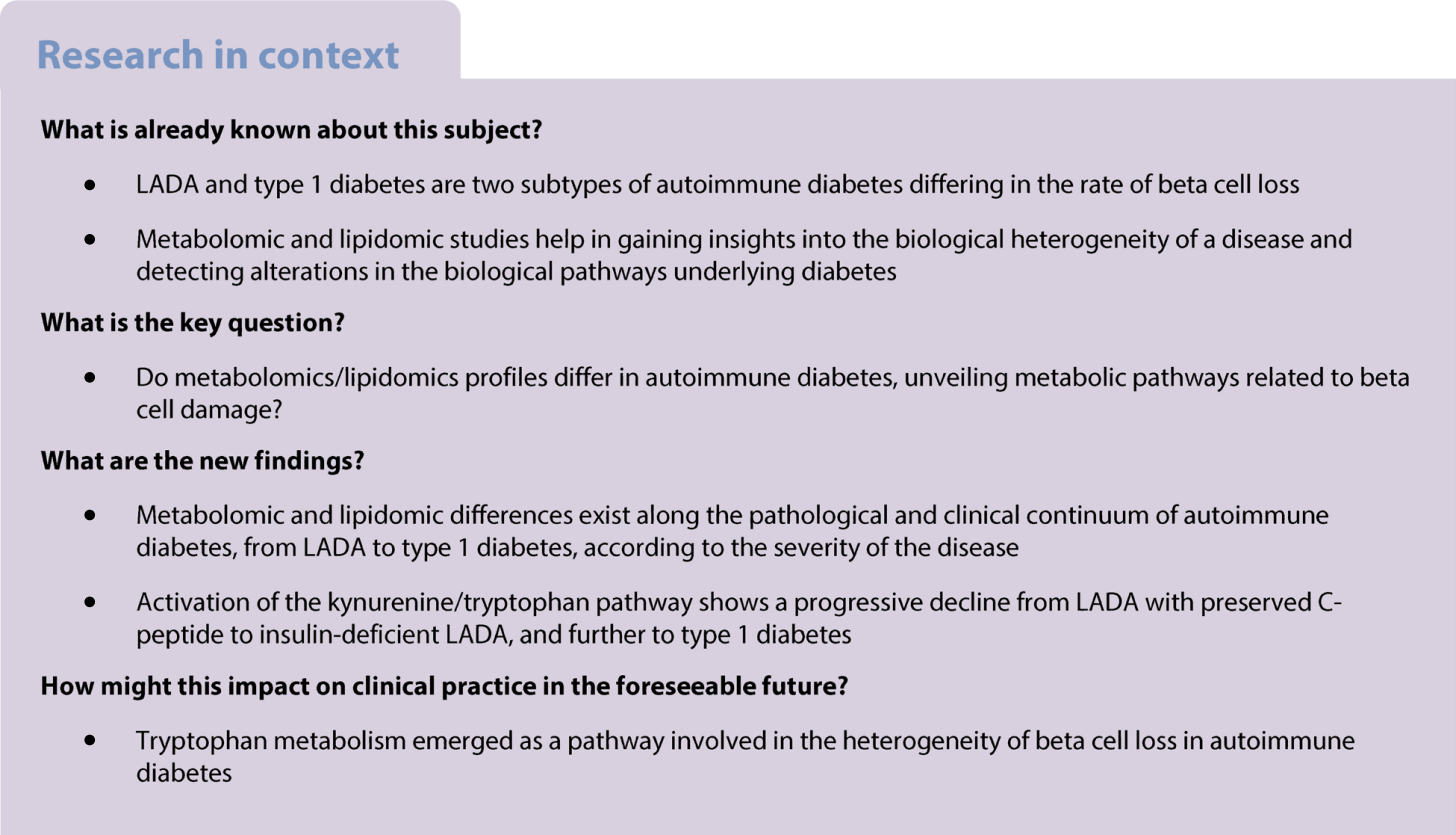


## Introduction

Autoimmune diabetes comprises a heterogeneous group of disorders characterised by progressive loss of endogenous insulin secretion due to immune-mediated destruction of pancreatic beta cells, ultimately leading to hyperglycaemia [1]. Differences in the rate of beta cell loss among subtypes of autoimmune diabetes have been recognised for decades [2]. Some individuals experience rapid progression to insulin dependence, often coinciding with disease onset, and are diagnosed with type 1 diabetes; others remain insulin-independent for months or years after diagnosis [3]. Compared with the former, these individuals are more often diagnosed in adulthood, show milder metabolic decompensation at diagnosis, and exhibit a slower decline of beta cell function over time [4]. These patients are classified as having a less severe form of autoimmune diabetes, named latent autoimmune diabetes in adults (LADA) [5, 6]. Characterising differences between type 1 diabetes and LADA may help identify biological pathways involved in the fast vs slow beta cell loss. As disease heterogeneity likely encompasses interactions between genetic and environmental factors, research methods allowing an integrated understanding of complex biological pathways are needed [7]. In this regard, metabolomic and lipidomic analyses allow the non-invasive study of circulating metabolites and lipids, providing a functional readout of chemical fingerprints, informing about the cellular processes taking place and disease-related alterations [8]. Given the different progression of beta cell loss in type 1 diabetes and LADA, we hypothesised that immune-related mechanisms of beta cell destruction and protection could be reflected in differences in their metabolomic profile, particularly in metabolites related to glycolysis and the tricarboxylic acid (TCA) cycle, proinflammatory lipids such as ceramides (CERs) and sphingolipids, and amino acids involved in inflammation like tryptophan and its metabolites. In this study our aim was to compare the metabolomic and lipidomic profile of people with LADA and type 1 diabetes, using healthy control participants and people with rheumatoid arthritis (RA) – an autoimmune disease that shares common features with autoimmune diabetes – as reference groups, to identify metabolic markers specifically linked to diabetes and diabetic autoimmunity. Metabolomic and lipidomic changes of in vitro models of beta cell damage were also evaluated to further support the biological plausibility of the systemic findings.

## Methods

### Study design and population

Five study centres operating in two different regions of Italy (Lazio and Tuscany) collaborated to conduct this cross-sectional study: Unit of Diabetes, Umberto I General Hospital (Sapienza University of Rome); Unit of Diabetes and Metabolic Disease, University of Siena; Department of Experimental and Clinical Medicine, University of Pisa; Institute of Clinical Physiology, National Research Council, Pisa; Unit of Immuno-rheumatology, Fondazione Policlinico Universitario Campus Bio-medico, Rome. Participants were recruited from a predominantly European ancestry population. People with a diagnosis of LADA or with a diagnosis of type 1 diabetes attending the study centres were consecutively enrolled. The diagnosis of LADA was performed according to the criteria proposed by the Immunology of Diabetes Society [9]. Type 1 diabetes was diagnosed according to the criteria proposed by the American Diabetes Association [5]. To identify immune pathways specifically related to diabetic autoimmunity, people with a diagnosis of RA, without diabetes, were also enrolled as a reference group of people with a non-diabetic autoimmune disorder [10]. People with a confirmed diagnosis of familial hypercholesterolaemia, active malignancy, other forms of diabetes and pregnant women were excluded from the study. We enrolled both male and female participants without any specific restriction related to sex.

Metabolomic data from the study cohort were compared with averaged data from individuals without diabetes or other comorbidities – the control group – derived from an anonymised database generated from previously characterised populations, analysed with the same analytical method. Data were selected based on the availability of kynurenine/tryptophan (Kyn/Trp) measurements within the metabolomic profile, as well as anthropometric and clinical parameters comparable to those collected in the present study

### Ethics

The study was performed in accordance with the Declaration of Helsinki, and the protocol was approved by the ethical committee of Policlinico Umberto I General Hospital (the coordinating centre) and by the ethical committees of satellite centres (Prot. 287/2020, ref 5984). Participants gave informed consent for participating in the study.

### Clinical data and biochemistry

Blood samples were collected at the study centres in the morning, following an overnight fast of at least 10 h. Participants on insulin were instructed to inject the last dose of basal insulin on the evening prior to blood sampling and to refrain from injecting short-acting insulin within the 5 h preceding venepuncture. Further details are reported in the electronic supplementary material (ESM) Methods.

Age, sex, body mass index (BMI, kg/m^2^), fasting blood glucose, HbA_1c_ (not available for people with RA), lipid profile (total cholesterol, HDL-cholesterol, triglycerides, LDL-cholesterol) and serum creatinine (not available for control participants) were retrieved from electronic medical records. Sex was recorded as male or female (biological sex), while gender identity information was not collected. Estimated glomerular filtration rate (eGFR) was calculated using the EPI-CKD formula [11].

Fasting C-peptide was measured only in people with LADA by immunoradiometric assay (IRMA) using a commercial C-peptide IRMA kit (Pantec, Prague, Czech Republic; Catalogue no. IM3639).

Serum concentrations of alanine aminotransferase (ALT), aspartate aminotransferase (AST), gamma-glutamyl transferase (GGT), uric acid, urea, and non-esterified fatty acids (NEFA) were quantified centrally by standard spectrophotometric methods using the Daytona instrument (Randox Laboratories, Crumlin, UK).

### Lipidomic and metabolomic profiling

The lipidomic and metabolomic profile included circulating lipids and metabolites (see ESM Methods). Briefly, lipidomics was performed at the Multi-Omics Mass Spectrometry laboratory of the Institute of Clinical Physiology, National Research Council, Pisa, by liquid chromatography high resolution mass spectrometry (UHPLC-MS QTOF, Agilent) in positive mode with untargeted acquisition and targeted quantification of lipids as previously described [12, 13]. Our targeted libraries contain more than 200 lipids belonging to seven different lipid classes (phosphatidylcholines [PCs], phosphatidylethanolamines [PEs], lysophosphatidylcholines [LPCs], lysophosphatidylethanolamines [LPEs], triacylglycerols [TAGs], sphingomyelins [SMs] and CERs) that have been previously implicated in alterations of glucose and lipid metabolism and inflammation. Lipids with spectral areas above the limit of detection (LOD) and a good signal-to-noise ratio were quantified using internal standards (Avanti Polar Lipids, Merck, Germany and Larodan, Sweden) [12]. Targeted analysis of plasma polar metabolites was also performed by gas chromatography tandem mass spectrometry (GC-MS/MS, Agilent) or UHPLC-MS QTOF and quantified using labelled internal standards (Cambridge Isotope Laboratories, MA, USA) [13]. We focused on amino acids and metabolites that are intermediate products of glycolysis, TCA cycle and tryptophan metabolism, where the Kyn/Trp is often used as an indicator of pathway activation [14].

### Human islets and in vitro experiments

Human islets were prepared by collagenase digestion and density gradient purification [15] from the pancreases of five organ donors without diabetes (two females/three males, age: median 72 years [25th–75th percentile: 55.5–78.5], BMI: 23.1 kg/m^2^ [22.9–34.1], cause of death: five [all] cardiovascular disease [CVD]; further details can be found in the human islets checklist in ESM). The organs were handled in Pisa before 30 November 2021, and processed with permission of the Ethics Committee of the University of Pisa, upon written consent of donors’ next of kin (21 November 2013, no. 2615). Islets were allowed to recover from isolation stress for 48 h in M199 medium (Euroclone SpA, Milan, Italy) containing 5.5 mmol/l glucose, supplemented with 10% (vol./vol.) adult bovine serum, 100 U/ml penicillin, 100 μg/ml streptomycin, 50 μg/ml gentamicin, and 750 ng/ml amphotericin B (all from Sigma-Aldrich, St Louis, MO, USA) at 37°C in a CO_2_ incubator. Approximately 500 islets were cultured in the same medium with or without 50 U/ml interleukin-1β (IL-1β) plus 1000 U/ml interferon-γ (IFN-γ, Sigma-Aldrich) for 48 h. Islets were then centrifuged at 400 *g* for 3 min, washed with ice-cold PBS, collected after further centrifugation at 3000 *g* for 5 min, snap-frozen as dry pellet and stored at −80°C till metabolomics/lipidomics analyses (details in ESM Methods).

### Statistical analysis

Data are presented as numbers with proportions for categorical variables, and as median [25th–75th percentile] for continuous variables.

Group differences in Table 1 were analysed by Kruskal–Wallis with post hoc Mann–Whitney *U* tests. Categorical variables were compared using χ^2^ or Fisher’s exact tests, as appropriate. Metabolomic and lipidomic profiles across LADA, type 1 diabetes, RA and control groups were visualised using heatmaps (scaled to zero mean and unit variance; group means displayed). Colours indicate relative abundance (red, higher; blue, lower; grey, no change). *p* values were adjusted for false discovery rate (FDR) using the Benjamini–Hochberg procedure [16], with FDR-adjusted *p*<0.05 considered significant. Variables with >35% missing data and outliers exceeding ±3 SD were excluded.

**Table 1:** Population features

Characteristic	T1D	LADA	RA	CT	*p* value	LADA vs T1D	LADA vs RA	T1D vs RA	CT vs LADA	CT vs T1D	CT vs RA
*n*	44	49	29	14							
Sex (male/female)	19/25	17/32	8/21	6/8	0.55	0.52	0.22	0.62	0.75	1.00	0.49
Age, years	41.0 [29.0–53.0]	55.5 [50.8–60.3]	59.4 [53.1–68.1]	46.0 [34.0–51.8]	0.05	<0.0001	0.04	<0.0001	0.003	0.39	0.0004
Age at diabetes diagnosis, years	20.0 [15.0–27.0]	47.5 [41.0–54.5]	N/A	N/A	N/A	<0.0001	N/A	N/A	N/A	N/A	N/A
Diabetes duration, years	18.0 [11.8–23.0]	5.0 [3.0–11.0]	N/A	N/A	N/A	<0.0001	N/A	N/A	N/A	N/A	N/A
BMI, kg/m^2^	24.0 [21.4–26.2]	25.1 [23.4–27.9]	25.6 [24.2–26.5]	29.1 [24.1–30.5]	0.08	0.13	0.93	0.26	0.39	0.03	0.53
HbA_1c_, mmol/mol	58.5 [53.0–67.2]	58.5 [46.5–68.3]	N/A	35.5 [35.5–39.9]	0.005	0.81	N/A	N/A	0.003	0.002	N/A
HbA_1c_, %	7.5 [7.0–8.3]	7.5 [6.4–8.4]	N/A	5.4 [5.4–5.8]	0.005	0.81	N/A	N/A	0.003	0.002	N/A
eGFR, ml/min per 1.73m^2^	73.0 [61.0–86.0]	89.0 [76.0–105.0]	84.0 [72.0–99.0]	N/A	<0.0001	<0.0001	0.27	0.026	N/A	N/A	N/A
Fasting blood glucose, mmol/l	8.7 [7.0–11.9]	8.4 [6.4–12.0]	5.5 [5.0–6.3]	5.3 [4.6–5.4]	<0.0001	0.66	<0.0001	<0.0001	<0.0001	<0.0001	0.33
Total cholesterol, mmol/l	4.7 [4.2–5.4]	4.6 [4.2–5.3]	4.6 [4.1–5.6]	4.8 [3.9–5.0]	0.93	0.81	0.93	0.92	0.77	0.76	0.76
HDL-cholesterol, mmol/l	1.7 [1.4–2.0]	1.6 [1.4–2.0]	1.7 [1.3–1.9]	1.2 [1.1–1.4]	0.03	0.66	0.93	0.82	0.02	0.004	0.19
LDL-cholesterol, mmol/l	2.5 [2.0–2.7]	2.6 [2.1–3.0]	2.5 [1.9–3.2]	2.7 [1.9–3.1]	0.69	0.42	0.93	0.93	0.93	0.5	0.79
Triglycerides, mmol/l	1.2 [0.7–1.7]	0.8 [0.6–1.1]	1.1 [0.8–1.6]	1.1 [0.6–1.3]	0.05	0.05	0.04	0.92	0.44	0.59	0.76
Creatinine, μmol/l	88.4 [79.6–99.9]	69.0 [56.6–79.6]	71.6 [58.3–79.6]	N/A	<0.0001	<0.0001	0.93	<0.0001	N/A	N/A	N/A
AST, U/l	20.9 [18.7–24.5]	21.2 [18.4–28.3]	24.0 [21.2–26.8]	20.0 [18.0–26.0]	0.82	0.74	0.93	0.54	0.77	0.89	0.76
ALT, U/l	18.0 [19.0–27.9]	25.6 [18.9–37.8]	17.5 [14.5–25.0]	16.0 [15.3–24.3]	0.02	0.03	0.04	0.92	0.08	0.89	0.79
GGT, U/l	24.0 [15.6–29.3]	20.7 [15.4–35.2]	23.6 [15.0–31.7]	17.0 [13.3–26.5]	0.63	0.66	0.93	0.92	0.27	0.47	0.53
Urea, mmol/l	5.7 [4.9–6.7]	6.4 [5.6–7.2]	5.6 [5.0–7.6]	4.8 [4.1–5.1]	0.16	0.45	0.93	0.92	0.07	0.08	0.33
Uric acid, mmol/l	0.22 [0.18–0.28]	0.23 [0.29–0.54]	0.24 [0.22–0.33]	N/A	0.19	0.77	0.15	0.16	N/A	N/A	N/A
NEFA, mmol/l	0.34 [0.23–0.60]	0.43 [0.30–0.81]	0.59 [0.46–1.03]	0.59 [0.45–0.76]	0.002	0.12	0.08	<0.0001	0.39	0.02	0.76
Statins, *n* (%)	16.0 (36.4)	27.0 (55.1)	5.0 (17.2)	N/A	0.01	0.16	0.009	0.21	N/A	N/A	N/A

Data are reported as median [25th–75th percentile] for continuous variables and as numbers (%) for categorical variables

Kruskal–Wallis and Mann–Whitney *U* test *p* values are reported for overall and pairwise comparisons of continuous variables, respectively

χ^2^ test *p* values are reported for categorical variables

All *p* values are adjusted for false discovery rate

CT, control; N/A, not available; T1D, type 1 diabetes

Multivariable regression was used to assess the association between Kyn/Trp and C-peptide (*n*=41 LADA) adjusting for prespecified confounders known to potentially impact beta cell function or C-peptide clearance: diabetes duration, age, sex, BMI, eGFR, HbA_1c_, insulin therapy [17]. Analysis of covariance (ANCOVA) evaluated group differences (LADA, type 1 diabetes, RA) normalised to controls, adjusting for diabetes duration, age, sex and eGFR. Covariates were retained if associated with the outcome at a conservative *p*<0.1 in univariate analyses. Non-normally distributed variables were log-transformed prior to regression and ANCOVA.

Islet experiment data were expressed as log_2_ fold change (stimulated with IL-1β and IFN-γ vs non-stimulated; pmol/million islets). Paired *t* tests were performed on log-transformed data. Raw and FDR-adjusted *p* values (Benjamini–Hochberg) are reported.

Statistical analyses were performed using R, STATA/IC 11 (StataCorp), and Prism 10.0 (GraphPad).

## Results

### Population features

A total of 136 participants were enrolled in this study: 44 with type 1 diabetes, 49 with LADA, 29 with RA, and 14 control participants. Population features are summarised in Table 1. Briefly, participants in the control and type 1 diabetes groups were slightly younger (mean age 46 and 41 years, respectively) than those in the RA and LADA groups (59 and 56 years, respectively). Compared with type 1 diabetes, individuals with LADA had a shorter disease duration and higher eGFR, while HbA_1c_ levels and sex distribution were comparable between the two groups. Although participants with RA and LADA were older than control participants, age stratification (<45 vs ≥45 years) did not reveal significant differences in metabolomic profiles. As expected, HbA_1c_ and fasting glucose levels were higher in the LADA and type 1 diabetes groups compared to the control group. HDL-cholesterol levels were also higher in both groups, although remaining within the normal range. In contrast, BMI was lower in type 1 diabetes compared with the control group, despite a wide BMI distribution in the control group. All individuals with type 1 diabetes and the majority of those with LADA (41/49, 83.7%) were receiving insulin therapy; among these, 85.4% were on intensive insulin regimens, defined as ≥3 daily injections or continuous subcutaneous insulin infusion. Use of additional glucose-lowering therapies in the LADA group is reported in ESM Table 1.

### Lipidomics and metabolomics profiling

**Fig. 1:**
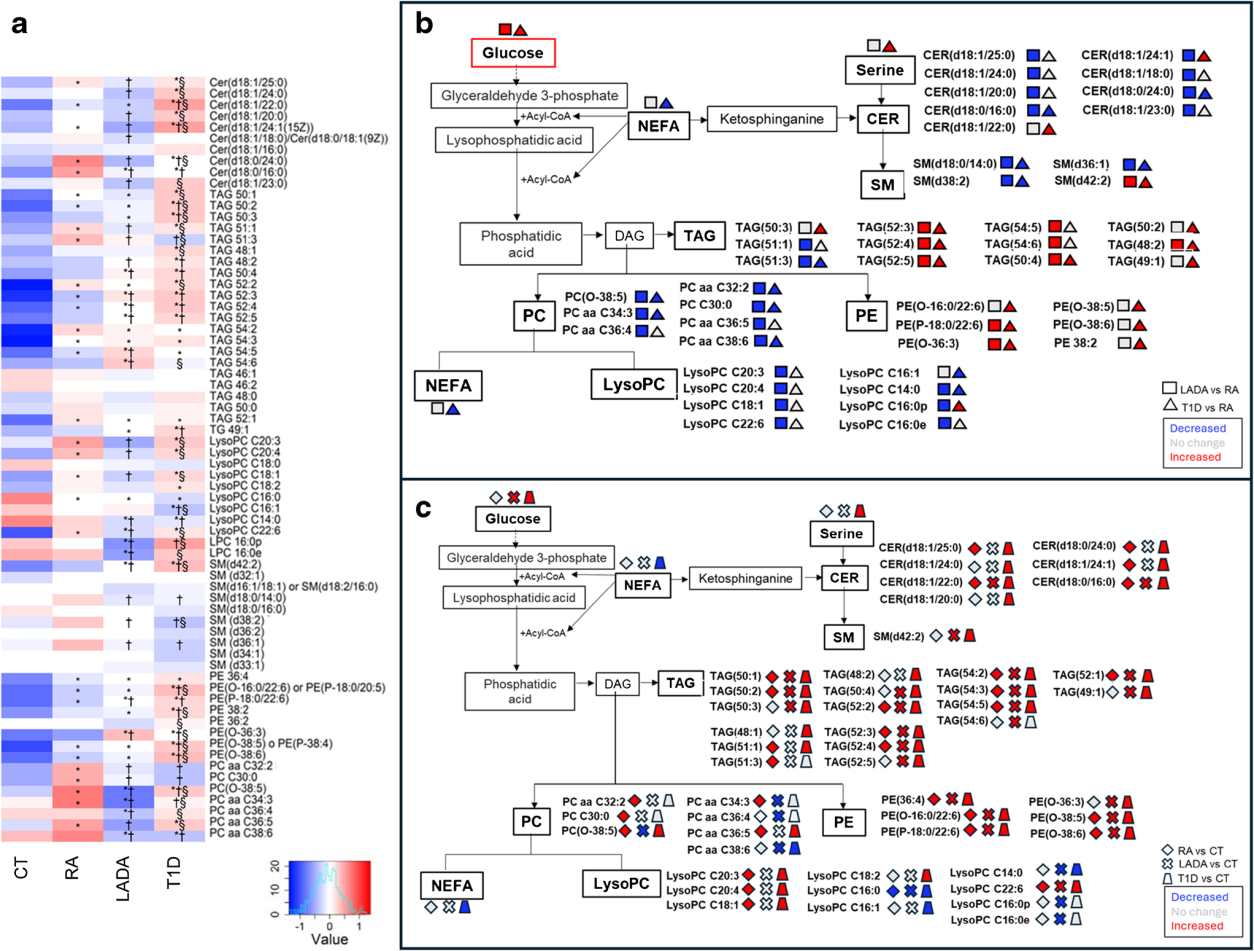
Lipidomic profile in the disease groups: LADA, T1D and RA, and control (CT). (**a**) Heatmap of lipids in the four groups. Data were scaled to zero mean and unit variance and reported as mean within the four groups. Post hoc *t* test *p* value after controlling for confounding variables (sex, age, BMI) and adjusted for FDR: *p<0.05 vs CT, ^†^*p*<0.05 vs RA, ^§^*p*<0.05 vs LADA. (**b**) and (**c**) report the lipid pathway and the lipid species which significantly differed between groups. (**b**) Comparison between T1D vs RA and LADA vs RA; (**c**) comparison between T1D vs CT, LADA vs CT and RA vs CT. The colour legend in (**b**, **c**) indicates relative concentrations: higher (red), lower (blue) and no change (grey). DAG, diacylglycerols; PE, O-phosphatidylethanolamine, T1D, type 1 diabetes

**Fig. 2:**
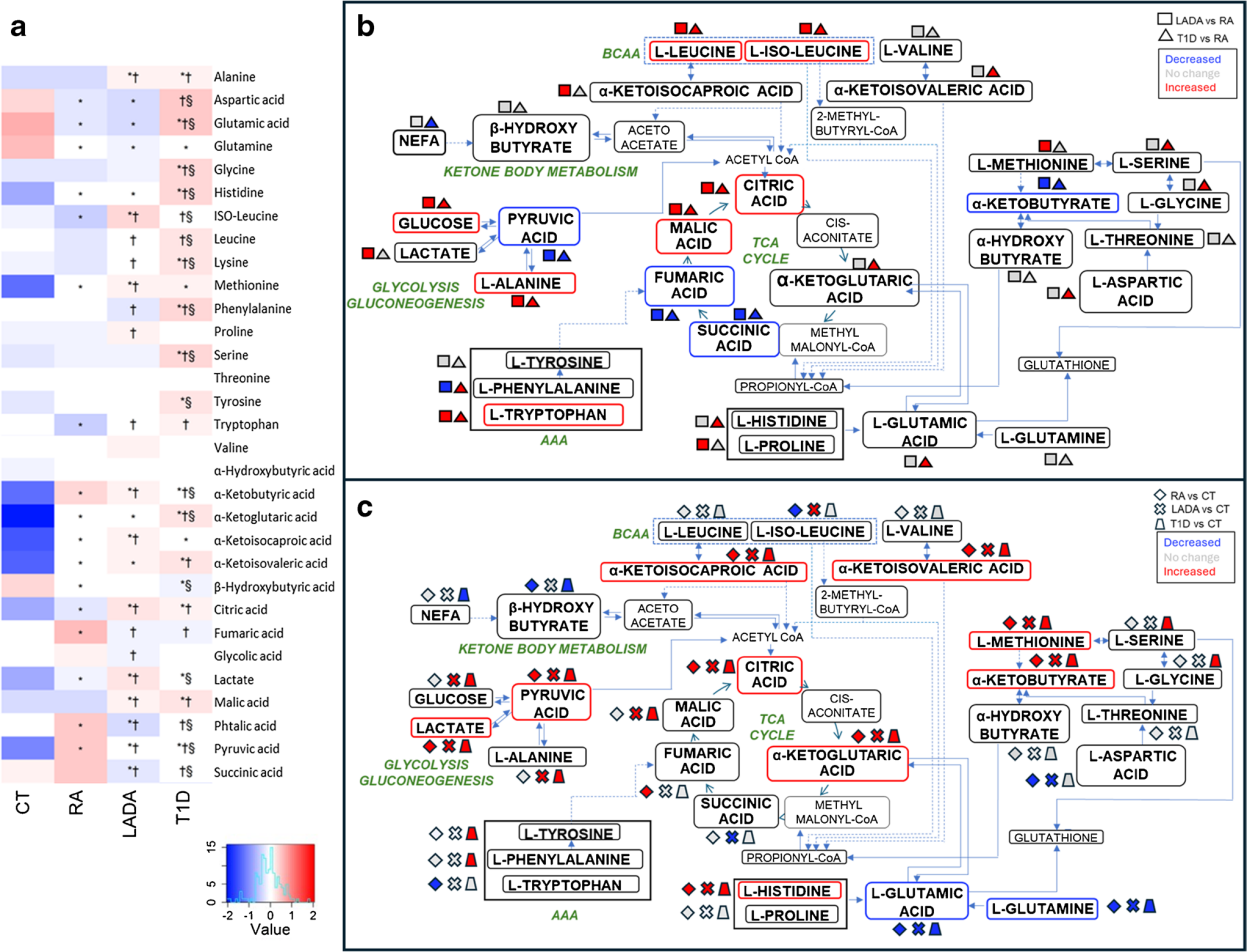
Concentrations of amino acids and metabolites involved in glucose metabolism in the disease groups: LADA, T1D and RA, and control (CT). (**a**) Heatmap of polar metabolites in the four groups. Data were scaled to zero mean and unit variance and reported as mean within the four groups. Post hoc *t* test *p* value after controlling for confounding variables (sex, age, BMI) and adjusted for FDR: *p<0.05 vs CT, ^†^*p*<0.05 vs RA, ^§^*p*<0.05 vs LADA. (**b**) and (**c**) report the most important amino acids, polar metabolites of the TCA cycle and glycolysis that differed between groups. (**b**) Comparison between T1D vs RA and LADA vs RA; (**c**) comparison between T1D vs CT, LADA vs CT and RA vs CT. The colour legend in (**b**, **c**) indicates relative concentrations: higher (red), lower (blue) and no change (grey). Solid lines indicate direct reactions, whereas dotted lines indicate indirect connections via omitted intermediates. AAA, aromatic amino acids; BCAA, branched-chain amino acids; T1D, type 1 diabetes

Significant differences in the levels of several metabolites and lipids were observed across the groups. Compared with the control group, most unsaturated TAGs and PEs, except PE 36:2, were elevated across all disease groups, with the highest levels observed in type 1 diabetes. CER levels were also higher in type 1 diabetes, whereas widespread elevations in PCs were primarily observed in RA (Fig. 1a, b; ESM Table 2). When comparing disease groups, several CERs, PCs and LPCs were significantly lower in LADA than in both type 1 diabetes and RA, while differences between type 1 diabetes and RA were generally less pronounced for these lipids. In contrast, SM(d42:2) and O-PE species were higher in type 1 diabetes compared with LADA and RA. Additionally, PCaa C32:2, C30:0, C38:6, as well as LPC C14:0, were lower in both type 1 diabetes and LADA compared with RA. In contrast, LPC C16:1 was lower in type 1 diabetes than LADA (Fig. 1c). Considering metabolomic analysis, most metabolites were elevated in autoimmune diseases compared with the control group, except for glutamate and glutamine (Fig. 2a, ESM Table 3). Significant differences were observed in polar metabolites involved in glycolysis and in the TCA cycle between autoimmune diabetes and RA (Fig. 2b). Succinate concentration was lower in LADA, whereas NEFA and β-hydroxybutyrate (BHB) levels were lower in type 1 diabetes (Fig. 2c).

The differences found among the four groups in metabolomic and lipidomic profiles did not change after stratification according to BMI (≤27 kg/m^2^ and >27 kg/m^2^) (ESM Tables 4, 5).

**Fig. 3:**
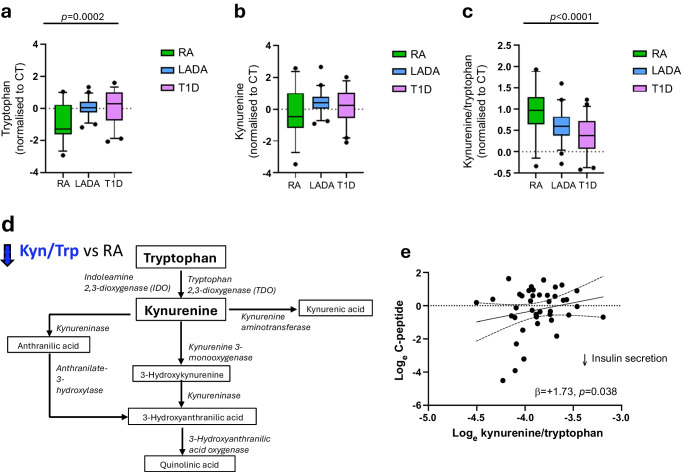
Kyn/Trp ratio in autoimmune diabetes. (**a**–**c**) Tryptophan, kynurenine and Kyn/Trp ratios in individuals with T1D (*n*=44), LADA (*n*=49), RA (*n*=29). Values were normalised to the median of CT and log_2_-scaled. Data are presented as box-and-whisker plots, where the central line represents the median, the box represents the interquartile range (25th–75th percentiles), and the whiskers indicate the 5th and 95th percentiles. ANCOVA *p* values using sex, age, and BMI, and after adjusting for FDR, refer to the overall comparison among the three groups. (**d**) illustrates the tryptophan metabolic pathways. (**e**) shows the positive correlation between C-peptide and Kyn/Trp, among people with LADA; beta-coefficient and *p* value are adjusted for confounders as detailed in the main text. CT, control; T1D, type 1 diabetes

When looking at differences between LADA and type 1 diabetes, type 1 diabetes was characterised by higher values of most amino acids, except iso-leucine, and metabolites at the main entry points of the TCA cycle, such as glutamate, alpha-ketoglutarate and succinate (Fig. 2a); on the other hand, higher levels of lactate were observed in individuals with LADA compared to those with type 1 diabetes, whereas pyruvate, NEFA and BHB levels were lower in type 1 diabetes [14]. The differences found between LADA and type 1 diabetes remained significant after adjustment for sex, age, BMI, HbA_1c_, insulin and statin use (ESM Tables 6–9). Levels of alanine, methionine and tryptophan were higher in autoimmune diabetes compared with RA, without differences between LADA and type 1 diabetes (Figs 2, 3).

The incubation of human islets with cytokines, which mimics the autoimmune response, showed that only few metabolites and lipids were changed (ESM Figs 1, 2; ESM Tables 10, 11). Among these, we observed a decrease in aspartic acid (by −43%) and a strong decrease in tryptophan (by −80%) [14]. No correlation was observed between donor age and islet function.

### Kynurenine/tryptophan

Based on the results of the islet metabolome profiling, and because of the described involvement of tryptophan metabolism in immunomodulatory pathways [14], including in pancreatic islets [18, 19], we then focused our analysis on tryptophan, kynurenine and the Kyn/Trp ratio. We found reduced tryptophan concentrations in RA, while kynurenine concentrations were similar across the autoimmune disease groups (ESM Table 3, Fig. 3a, b). Specifically, people with LADA or type 1 diabetes showed a higher Kyn/Trp ratio (median [25th–75th percentile]) (0.021 [0.018–0.024] and 0.018 [0.014–0.022], respectively) when compared with control participants (0.014 [0.012–0.019]), but lower when compared to people with RA (0.027 [0.022–0.032]) (Fig. 3c). Differences among the groups remained statistically significant also after adjustments for sex, age and eGFR (*p*=0.010 for type 1 diabetes vs LADA; *p*=0.015 for LADA vs RA; *p*<0.001 for type 1 diabetes vs RA). The significant trend of decreasing Kyn/Trp ratio from RA to LADA to type 1 diabetes was also maintained after normalisation to control median values and adjustment for other confounders indicating a reduced tryptophan metabolism (Fig. 3d). Within the group of people with autoimmune diabetes, those with type 1 diabetes had lower Kyn/Trp ratio compared to LADA also after additional adjustment for diabetes duration (*p*=0.0079). However, the difference between LADA and type 1 diabetes was no longer statistically significant after adjusting for BMI, although BMI was similar in the two groups (Table 1).

Serum C-peptide concentrations in people with LADA were directly related to Kyn/Trp ratio, independently from confounders, including duration of disease (Fig. 3e). In LADA, insulin-deficient participants (C-peptide <0.3 nmol/l) had lower Kyn/Trp values than non-insulin-deficient individuals (median [25th–75th percentile]) (0.0173 [0.0165–0.0181] vs 0.0209 [0.0191–0.0250]; *p* value 0.022 adjusted for diabetes duration) and showed values comparable to type 1 diabetes.

## Discussion

This study describes the metabolomic and lipidomic profiles of autoimmune diabetes along its pathological continuum, showing differences between type 1 diabetes and LADA, both of which exhibit alterations when compared with control groups of people without diabetes or affected by RA, a non-diabetic autoimmune condition sharing many factors with autoimmune diabetes. Such differences are mainly observed in specific lipidomic species, polar metabolites involved in glucose and tryptophan metabolism.

### Metabolomic heterogeneity in autoimmune diabetes

Since diabetes is characterised by variable metabolic dysfunctions, a different metabolomics profile might be expected when comparing autoimmune diabetes with RA, similar to type 1 diabetes in terms of genetic susceptibility, some pathogenetic pathways and clinical course [20–22]. Compared with LADA, type 1 diabetes showed differences in metabolite and lipid concentrations which remained significant after adjustment for confounding variables, suggesting that the recognised heterogeneity of adult-onset autoimmune diabetes extends to several physiological and biochemical processes differing among subtypes of the disease even years after diabetes onset.

In contrast to our study, most previous metabolomic evaluations in type 1 diabetes were conducted in children at preclinical stages of the disease, aiming to characterise metabolic pathways that might inform strategies to prevent or delay the clinical onset of the disease [23–26]. These studies were largely untargeted, without absolute quantification of metabolites or lipids. In contrast, our study was focused on adults with overt autoimmune diabetes, using fully quantitative metabolomics/lipidomics analyses to explore metabolic differences between type 1 diabetes and LADA, two subtypes of the same disease that differ in the rate of beta cell loss [27], and in comparison with adults with another autoimmune disease without diabetes, RA, as well as healthy control participants.

Previous metabolomic analyses of LADA were limited to a selected subpopulation of the ANDIS cohort [28], also showing that LADA exhibits a different metabolic profile compared to type 1 diabetes, with all lipids and polar metabolites lower in type 1 diabetes. However, in the ANDIS cohort, type 1 diabetes participants had markedly higher HbA_1c_ levels (mean HbA_1c_ 100 mmol/mol [11.3%] vs 64 mmol/mol [8.0%] in LADA), lower BMI (21.0 kg/m^2^ vs 28.3 kg/m^2^) and a higher proportion of female participants, which could confound comparisons. In our study, type 1 diabetes and LADA participants had similar HbA_1c_ (58 mmol/mol [7.5%], Table 1), BMI (24.0 kg/m^2^ vs 25.1 kg/m^2^), sex distribution and cholesterol levels, reducing the likelihood that these factors influenced the observed metabolic differences.

### Lipidomics in LADA, type 1 diabetes and RA

People with autoimmune diabetes have different triacylglycerol levels compared with both RA and control groups, which, consistent with previous findings [29–31], might be the result of the exogenous insulin therapy through the subcutaneous route, the suboptimal glycaemic management of the enrolled population, or the different dietary habits. The observed differences in sphingolipids are consistent with previous findings of low SM levels observed among children progressing to, or with newly diagnosed type 1 diabetes compared to control participants [24, 32], and studies which demonstrated the metabolic reprogramming driven by chronic inflammation and the progression of autoimmunity [33].

PEs also differed between autoimmune diabetes (both type 1 diabetes and LADA) and RA and the control group, which again agrees with previous observations comparing children or newly diagnosed young adults with type 1 diabetes with control participants [32]. Notably, in our study people with type 1 diabetes showed even higher PE levels when compared with LADA, highlighting a possible role for this species also in the different severity of autoimmune diabetes. Moreover, LADA showed lower levels of CERs and LPCs, both species with known roles in the regulation of the immune system [34, 35]. Similarly, alteration in LPCs have been found to be associated with progression to type 1 diabetes in children with autoantibody against GAD (GADA-first) before 21 months of age [36].

### Polar metabolites in LADA, type 1 diabetes, and RA

The concentration of polar metabolites also differed between autoimmune diabetes and RA with generally higher levels observed in LADA and type 1 diabetes relative to the control group, suggesting altered glucose metabolism in individuals with autoimmune diabetes compared with participants without diabetes. Notably, both LADA and type 1 diabetes exhibited higher levels of TCA cycle metabolites compared with the control group, with the exception of succinate, which was lower in LADA. This finding aligns with recent evidence suggesting that succinate may function as an immunometabolite, modulating inflammatory signalling and immune responses in autoimmune diabetes [37]. Moreover, individuals with type 1 diabetes showed significantly higher concentrations of most amino acids and metabolites entering the TCA cycle compared with those with LADA. These findings suggest that TCA cycle metabolism may differ between LADA and type 1 diabetes, contributing not only to distinct alterations in glucose metabolism but also to perturbations in interconnected biochemical pathways, such as glutamine–glutamate and glutathione metabolism. Such disruptions may have downstream effects on redox homeostasis, amino acid metabolism and protein synthesis, potentially influencing disease pathophysiology and immune function in autoimmune diabetes relative to RA and healthy control participants.

### Tryptophan metabolism and Kyn/Trp ratio

The in vitro experiments on healthy human islets showed that the tryptophan metabolism was activated upon exposure to cytokines which are known to induce beta cell damage [38].

Metabolomic analysis in serum of individuals with autoimmune disease revealed activation of the kynurenine pathway of tryptophan metabolism, not only in type 1 diabetes and LADA, but also in RA, consistent with previous reports in RA [39] and with the concept that inflammatory states induce tryptophan catabolism to promote immune tolerance [40]. However, our data suggest a relative impairment of this pathway in autoimmune diabetes, with progressively lower Kyn/Trp ratios from LADA with preserved C-peptide to insulin-deficient LADA and classical type 1 diabetes.

The metabolism of tryptophan via the kynurenine pathway was previously shown to be involved in the control of immune responses, and a dysregulation of such a pathway has been associated with autoimmune disorders [41, 42]. Indoleamine 2,3-dioxygenase-1 (IDO1) is the key enzyme expressed in immune cells responsible for the conversion of tryptophan in kynurenine, and impairment of IDO1 activity, resulting in higher tryptophan and reduced kynurenine, has been documented in animal models of autoimmune diabetes [43–45], as well as in children with type 1 diabetes [46].

Furthermore, a central role for the Kyn/Trp pathway has been documented also in the beta cell response to inflammation [18], as also suggested by our in vitro experiments. Additionally, IDO1 expression was found to be lower in beta cells from double autoantibody-positive donors and patients with recent-onset type 1 diabetes [19]. Our findings extend previous knowledge by demonstrating that the plasma Kyn/Trp ratio, a marker of systemic tryptophan metabolism, is reduced in adults with type 1 diabetes and in individuals with LADA who have a diminished beta cell reserve, and that this reduction correlates with reduced insulin secretion in adult-onset autoimmune diabetes.

### Strengths and limitations

This study’s strengths include its integrated approach, combining clinical, biochemical (serum metabolomic and lipidomic profiling) and human islet experiments to provide complementary insights into the heterogeneity of autoimmune diabetes. Targeted metabolomics and lipidomics allowed full quantification of serum lipids and metabolites, offering greater precision than untargeted approaches [47]. The autoimmune diabetes groups were well-matched for sex, BMI and HbA_1c_ values, and by including control groups both with and without a non-diabetic autoimmune disease, our study provides reference metabolite levels, allowing a more comprehensive understanding of the metabolic alterations specific to autoimmune diabetes. In our study the number of male and female participants was balanced among study groups, which supports generalisability across sexes. Limitations include the cross-sectional study design and the absence of C-peptide values in type 1 diabetes, which we assume to be low because of the clinical course of the disease and the early insulin requirement [48]. Nonetheless, our data support the need for future studies evaluating whether the investigated biological pathways might be implicated in the preservation of C-peptide micro-secretions among people with classical type 1 diabetes. Furthermore, serum Kyn/Trp ratio does not necessarily reflect IDO1 activity, as systemic tryptophan metabolism is also regulated by other enzymes, particularly the hepatic tryptophan 2,3-dioxygenase (TDO), the main determinant of the systemic tryptophan levels under normal conditions. In this context, a longitudinal approach may provide additional clinical insights showing temporal changes in metabolic and immunological markers as suggested by previous studies [49]. Nonetheless, some evidence indicates that liver TDO expression is reduced during inflammation, favouring IDO1 activity in immune cells [41], and dysfunctional IDO1 activity was described in peripheral blood mononuclear cells from children with type 1 diabetes, showing lower serum Kyn/Trp ratio compared with the control group [46]. The study was not powered to detect sex-specific differences in outcomes, and sex-stratified analyses were not performed. The different therapeutic strategies used for type 1 diabetes and LADA might have influenced some metabolic pathway. However, insulin therapy was the main treatment used by the majority of LADA participants, and it was included as a covariate in the multivariate regression model evaluating the association between Kyn/Trp ratio and C-peptide.

Although SGLT2 inhibitors (SGLT2i) have been reported to increase BHB, this effect is mediated by elevated serum NEFA resulting from reduced insulin and increased glucagon, rather than by SGLT2i use per se [50]. Therefore, the higher BHB levels observed in LADA are unlikely driven by the small number of participants taking SGLT2i; indeed, even after excluding these individuals, LADA participants still exhibited significantly higher BHB levels than those with type 1 diabetes (data not shown).

Finally, we acknowledge that serum metabolites reflect systemic metabolism rather than islet-specific processes, and caution should be exercised when making inferences about islet pathophysiology, as such interpretations may be speculative.

### Conclusions

The metabolic signature of autoimmune diabetes subtypes characterised by different rates of beta cell loss has remained largely underexplored, with few targeted metabolomic investigations and no direct comparison with other non-diabetic autoimmune conditions. This study addresses this gap by providing new insights into the heterogeneity of autoimmune diabetes by identifying and quantifying distinct differences in lipid species and polar metabolites between the two main subtypes of the disease (type 1 diabetes and LADA).

Characterising these differences may help uncover the biological processes involved in the pathogenesis and progression of autoimmune diabetes, particularly in pathways related to beta cell damage and protection. By comparing groups with varying rates of progression towards an insulin-dependent state (type 1 diabetes and LADA), this study offers new insights.

In this regard, tryptophan metabolism has emerged as a key pathway involved in the severity of autoimmune diabetes, as evidenced by metabolomic data obtained from both human serum samples and from experiments on human beta cell cultures.

## Supplementary Information

Below is the link to the electronic supplementary material.

ESM (PDF 768 KB)

ESM (XLSX 73 KB)

## Data Availability

Supplementary material reports tables with lipids and metabolites measured for each group of subjects. Other data will be available for 5 years from the guarantors of the work upon reasonable request. Data will be shared for scientific research purposes only, subject to ethical approval and to the subscription of a data sharing agreement according to the institutional data sharing policy. Requests should be sent to the corresponding author(s), who will evaluate the appropriateness of the request.

## Abbreviations

ALT: Alanine aminotransferase
ANCOVA: Analysis of covariance
AST: Aspartate aminotransferase
BHB: β-hydroxybutyrate
BMI: Body mass index
CER: Ceramide
eGFR: Estimated glomerular filtration rate
FDR: False discovery rate
GGT: Gamma-glutamyl transferase
IDO1: Indoleamine 2,3-dioxygenase-1
Kyn/Trp: Kynurenine/tryptophan
LADA: Latent autoimmune diabetes in adults
LPC: Lysophosphatidylcholine
NEFA: Non-esterified fatty acids
PC: Phosphatidylcholine
PE: Phosphatidylethanolamine
RA: Rheumatoid arthritis
SM: Sphingomyelin
TAG: Triacylglycerol
TCA: Tricarboxylic acid

## References

[1] Katsarou A, Gudbjornsdottir S, Rawshani A et al (2017) Type 1 diabetes mellitus. Nat Rev Dis Primers 3:17016. 10.1038/nrdp.2017.16 28358037 10.1038/nrdp.2017.16

[2] Leslie RD, Kolb H, Schloot NC et al (2008) Diabetes classification: grey zones, sound and smoke: action LADA 1. Diabetes Metab Res Rev 24(7):511–519. 10.1002/dmrr.877 18615859 10.1002/dmrr.877

[3] Buzzetti R, Zampetti S, Maddaloni E (2017) Adult-onset autoimmune diabetes: current knowledge and implications for management. Nat Rev Endocrinol 13(11):674–686. 10.1038/nrendo.2017.99 28885622 10.1038/nrendo.2017.99

[4] Maddaloni E, Moretti C, Mignogna C, Buzzetti R (2020) Adult-onset autoimmune diabetes in 2020: an update. Maturitas 137:37–44. 10.1016/j.maturitas.2020.04.014 32498935 10.1016/j.maturitas.2020.04.014

[5] American Diabetes Association Professional Practice C (2024) 2. Diagnosis and classification of diabetes: standards of care in diabetes-2024. Diabetes Care 47(Suppl 1):S20–S42. 10.2337/dc24-S002 38078589 10.2337/dc24-S002 PMC10725812

[6] World Health Organization (2019) Classification of diabetes mellitus. Available from: https://www.who.int/publications/i/item/classification-of-diabetes-mellitus

[7] Sas KM, Karnovsky A, Michailidis G, Pennathur S (2015) Metabolomics and diabetes: analytical and computational approaches. Diabetes 64(3):718–732. 10.2337/db14-0509 25713200 10.2337/db14-0509 PMC4338589

[8] Johnson CH, Ivanisevic J, Siuzdak G (2016) Metabolomics: beyond biomarkers and towards mechanisms. Nat Rev Mol Cell Biol 17(7):451–459. 10.1038/nrm.2016.25 26979502 10.1038/nrm.2016.25 PMC5729912

[9] Fourlanos S, Dotta F, Greenbaum CJ et al (2005) Latent autoimmune diabetes in adults (LADA) should be less latent. Diabetologia 48(11):2206–2212. 10.1007/s00125-005-1960-7 16193284 10.1007/s00125-005-1960-7

[10] Xie W, Jiang H, Chen Y et al (2024) Relationship between type 1 diabetes and autoimmune diseases in european populations: a two-sample Mendelian randomization study. Front Genet 15:1335839. 10.3389/fgene.2024.1335839 39350769 10.3389/fgene.2024.1335839 PMC11439667

[11] Levey AS, Stevens LA, Schmid CH et al (2009) A new equation to estimate glomerular filtration rate. Ann Intern Med 150(9):604–612. 10.7326/0003-4819-150-9-200905050-00006 19414839 10.7326/0003-4819-150-9-200905050-00006 PMC2763564

[12] Della Pepa G, Carli F, Sabatini S et al (2024) Clusters of adipose tissue dysfunction in adults with type 2 diabetes identify those with worse lipidomic profile despite similar glycaemic control. Diabetes Metab Res Rev 40(4):e3798. 10.1002/dmrr.3798 38558269 10.1002/dmrr.3798

[13] Fenizia S, Scoditti E, Gastaldelli A (2023) Methods to study metabolomics. In: Federici M, Menghini R (eds) Gut microbiome, microbial metabolites and cardiometabolic risk. Springer International Publishing, Cham, pp 1–41

[14] Sorgdrager FJH, Naude PJW, Kema IP, Nollen EA, Deyn PP (2019) Tryptophan metabolism in inflammaging: from biomarker to therapeutic target. Front Immunol 10:2565. 10.3389/fimmu.2019.02565 31736978 10.3389/fimmu.2019.02565 PMC6833926

[15] Marselli L, Piron A, Suleiman M et al (2020) Persistent or transient human beta cell dysfunction induced by metabolic stress: specific signatures and shared gene expression with type 2 diabetes. Cell Rep 33(9):108466. 10.1016/j.celrep.2020.108466 33264613 10.1016/j.celrep.2020.108466

[16] Benjamini Y, Hochberg Y (1995) Controlling the false discovery rate: a practical and powerful approach to multiple testing. J R Stat Soc Ser B (Methodol) 57(1):289–300. 10.1111/j.2517-6161.1995.tb02031.x

[17] Maddaloni E, Bolli GB, Frier BM et al (2022) C-peptide determination in the diagnosis of type of diabetes and its management: a clinical perspective. Diabetes Obes Metab 24(10):1912–1926. 10.1111/dom.14785 35676794 10.1111/dom.14785 PMC9543865

[18] Liu JJ, Raynal S, Bailbe D et al (2015) Expression of the kynurenine pathway enzymes in the pancreatic islet cells. Activation by cytokines and glucolipotoxicity. Biochim Biophys Acta 1852(5):980–991. 10.1016/j.bbadis.2015.02.001 25675848 10.1016/j.bbadis.2015.02.001

[19] Anquetil F, Mondanelli G, Gonzalez N et al (2018) Loss of IDO1 expression from human pancreatic beta-cells precedes their destruction during the development of type 1 diabetes. Diabetes 67(9):1858–1866. 10.2337/db17-1281 29945890 10.2337/db17-1281 PMC6110313

[20] Newton JL, Harney SM, Wordsworth BP, Brown MA (2004) A review of the MHC genetics of rheumatoid arthritis. Genes Immun 5(3):151–157. 10.1038/sj.gene.6364045 14749714 10.1038/sj.gene.6364045

[21] Strollo R, Ponchel F, Malmstrom V et al (2013) Autoantibodies to posttranslationally modified type II collagen as potential biomarkers for rheumatoid arthritis. Arthritis Rheum 65(7):1702–1712. 10.1002/art.37964 23575908 10.1002/art.37964

[22] Strollo R, Rizzo P, Spoletini M et al (2013) HLA-dependent autoantibodies against post-translationally modified collagen type II in type 1 diabetes mellitus. Diabetologia 56(3):563–572. 10.1007/s00125-012-2780-1 23160643 10.1007/s00125-012-2780-1

[23] Pflueger M, Seppanen-Laakso T, Suortti T et al (2011) Age- and islet autoimmunity-associated differences in amino acid and lipid metabolites in children at risk for type 1 diabetes. Diabetes 60(11):2740–2747. 10.2337/db10-1652 22025777 10.2337/db10-1652 PMC3198092

[24] Lamichhane S, Ahonen L, Dyrlund TS et al (2018) Dynamics of plasma lipidome in progression to islet autoimmunity and type 1 diabetes - type 1 Diabetes Prediction and Prevention Study (DIPP). Sci Rep 8(1):10635. 10.1038/s41598-018-28907-8 30006587 10.1038/s41598-018-28907-8 PMC6045612

[25] Oresic M, Gopalacharyulu P, Mykkanen J et al (2013) Cord serum lipidome in prediction of islet autoimmunity and type 1 diabetes. Diabetes 62(9):3268–3274. 10.2337/db13-0159 23630305 10.2337/db13-0159 PMC3749353

[26] La Torre D, Seppanen-Laakso T, Larsson HE et al (2013) Decreased cord-blood phospholipids in young age-at-onset type 1 diabetes. Diabetes 62(11):3951–3956. 10.2337/db13-0215 23929934 10.2337/db13-0215 PMC3806611

[27] Buzzetti R, Maddaloni E, Gaglia J, Leslie RD, Wong FS, Boehm BO (2022) Adult-onset autoimmune diabetes. Nat Rev Dis Primers 8(1):63. 10.1038/s41572-022-00390-6 36138034 10.1038/s41572-022-00390-6

[28] Al-Majdoub M, Ali A, Storm P, Rosengren AH, Groop L, Spegel P (2017) Metabolite profiling of LADA challenges the view of a metabolically distinct subtype. Diabetes 66(4):806–814. 10.2337/db16-0779 27913577 10.2337/db16-0779

[29] Guy J, Ogden L, Wadwa RP et al (2009) Lipid and lipoprotein profiles in youth with and without type 1 diabetes: the SEARCH for Diabetes in Youth case-control study. Diabetes Care 32(3):416–420. 10.2337/dc08-1775 19092167 10.2337/dc08-1775 PMC2646019

[30] Verges B (2020) Dyslipidemia in type 1 diabetes: AMaskedDanger. Trends Endocrinol Metab 31(6):422–434. 10.1016/j.tem.2020.01.015 32217073 10.1016/j.tem.2020.01.015

[31] Gylling H, Tuominen JA, Koivisto VA, Miettinen TA (2004) Cholesterol metabolism in type 1 diabetes. Diabetes 53(9):2217–2222. 10.2337/diabetes.53.9.2217 15331530 10.2337/diabetes.53.9.2217

[32] Sorensen CM, Ding J, Zhang Q et al (2010) Perturbations in the lipid profile of individuals with newly diagnosed type 1 diabetes mellitus: lipidomics analysis of a Diabetes Antibody Standardization Program sample subset. Clin Biochem 43(12):948–956. 10.1016/j.clinbiochem.2010.04.075 20519132 10.1016/j.clinbiochem.2010.04.075 PMC2919299

[33] Andersson Svard A, Kaur S, Trost K et al (2020) Characterization of plasma lipidomics in adolescent subjects with increased risk for type 1 diabetes in the DiPiS cohort. Metabolomics 16(10):109. 10.1007/s11306-020-01730-x 33033923 10.1007/s11306-020-01730-x PMC7544716

[34] Lee M, Lee SY, Bae YS (2023) Functional roles of sphingolipids in immunity and their implication in disease. Exp Mol Med 55(6):1110–1130. 10.1038/s12276-023-01018-9 37258585 10.1038/s12276-023-01018-9 PMC10318102

[35] Kabarowski JH, Xu Y, Witte ON (2002) Lysophosphatidylcholine as a ligand for immunoregulation. Biochem Pharmacol 64(2):161–167. 10.1016/s0006-2952(02)01179-6 12123735 10.1016/s0006-2952(02)01179-6

[36] Li Q, Liu X, Yang J et al (2021) Plasma metabolome and circulating vitamins stratified onset age of an initial islet autoantibody and progression to type 1 diabetes: the TEDDY Study. Diabetes 70(1):282–292. 10.2337/db20-0696 33106256 10.2337/db20-0696 PMC7876562

[37] Harber KJ, de Goede KE, Verberk SGS et al (2020) Succinate is an inflammation-induced immunoregulatory metabolite in macrophages. Metabolites 10(9):372. 10.3390/metabo10090372 32942769 10.3390/metabo10090372 PMC7569821

[38] Ramos-Rodriguez M, Raurell-Vila H, Colli ML et al (2019) The impact of proinflammatory cytokines on the beta-cell regulatory landscape provides insights into the genetics of type 1 diabetes. Nat Genet 51(11):1588–1595. 10.1038/s41588-019-0524-6 31676868 10.1038/s41588-019-0524-6 PMC7040466

[39] Mangoni AA, Zinellu A (2023) A systematic review and meta-analysis of the kynurenine pathway of tryptophan metabolism in rheumatic diseases. Front Immunol 14:1257159. 10.3389/fimmu.2023.1257159 37936702 10.3389/fimmu.2023.1257159 PMC10626995

[40] Mellor AL, Munn DH (2004) IDO expression by dendritic cells: tolerance and tryptophan catabolism. Nat Rev Immunol 4(10):762–774. 10.1038/nri1457 15459668 10.1038/nri1457

[41] Krupa A, Kowalska I (2021) The kynurenine pathway-new linkage between innate and adaptive immunity in autoimmune endocrinopathies. Int J Mol Sci 22(18):9879. 10.3390/ijms22189879 34576041 10.3390/ijms22189879 PMC8469440

[42] Poormasjedi-Meibod MS, Jalili RB, Hosseini-Tabatabaei A, Hartwell R, Ghahary A (2013) Immuno-regulatory function of indoleamine 2,3 dioxygenase through modulation of innate immune responses. PloS one 8(8):e71044. 10.1371/journal.pone.0071044 23940687 10.1371/journal.pone.0071044 PMC3733714

[43] Zhang Y, Jalili RB, Kilani RT et al (2016) IDO-expressing fibroblasts protect islet beta cells from immunological attack and reverse hyperglycemia in non-obese diabetic mice. J Cell Physiol 231(9):1964–1973. 10.1002/jcp.25301 26743772 10.1002/jcp.25301

[44] Pallotta MT, Orabona C, Bianchi R et al (2014) Forced IDO1 expression in dendritic cells restores immunoregulatory signalling in autoimmune diabetes. J Cell Mol Med 18(10):2082–2091. 10.1111/jcmm.12360 25215657 10.1111/jcmm.12360 PMC4193887

[45] Fallarino F, Volpi C, Zelante T et al (2009) IDO mediates TLR9-driven protection from experimental autoimmune diabetes. J Immunol 183(10):6303–6312. 10.4049/jimmunol.0901577 19841163 10.4049/jimmunol.0901577

[46] Orabona C, Mondanelli G, Pallotta MT et al (2018) Deficiency of immunoregulatory indoleamine 2,3-dioxygenase 1in juvenile diabetes. JCI insight 3(6):e96244. 10.1172/jci.insight.96244 29563329 10.1172/jci.insight.96244 PMC5926942

[47] Sarkar S, Zheng X, Clair GC et al (2024) Exploring new frontiers in type 1 diabetes through advanced mass-spectrometry-based molecular measurements. Trends Mol Med 30(12):1137–1151. 10.1016/j.molmed.2024.07.009 39152082 10.1016/j.molmed.2024.07.009 PMC11631641

[48] Palmer JP, Fleming GA, Greenbaum CJ et al (2004) C-peptide is the appropriate outcome measure for type 1 diabetes clinical trials to preserve beta-cell function: report of an ADA workshop, 21–22 October 2001. Diabetes 53(1):250–264. 10.2337/diabetes.53.1.250 14693724 10.2337/diabetes.53.1.250

[49] Yan J, Kothur K, Mohammad S et al (2023) CSF neopterin, quinolinic acid and kynurenine/tryptophan ratio are biomarkers of active neuroinflammation. EBioMedicine 91:104589. 10.1016/j.ebiom.2023.104589 37119734 10.1016/j.ebiom.2023.104589 PMC10165192

[50] Abdelgani S, Khattab A, Adams J et al (2023) Distinct mechanisms responsible for the increase in glucose production and ketone formation caused by empagliflozin in T2DM patients. Diabetes Care 46(5):978–984. 10.2337/dc22-0885 36857415 10.2337/dc22-0885 PMC10154659

