# Supplementary material for: Metabolomic profile differs between LADA and type 1 diabetes identifying tryptophan metabolism as a pathway involved in the heterogeneity of autoimmune diabetes
Source: Diabetologia. 2026 Jun 26;69(9):2607–19. doi: 10.1007/s00125-026-06777-4 (PMC13423996; doi:10.1007/s00125-026-06777-4)
Supplement: Supplementary file 1 — ESM (PDF 768 KB) [file 125_2026_6777_MOESM1_ESM.pdf]

Electronic Supplementary Material (ESM) to: **Metabolomic profile differs between LADA and type 1 diabetes identifying tryptophan metabolism as a pathway involved in the heterogeneity of autoimmune diabetes.**

ESM index

ESM Methods

- Blood samples collection and processing________________________________Page 2
- Human Islets preparations and cytokine treatment______________________ Page 2
- Lipidomic analysis ____Page 3
- Metabolite analysis _______________ ___________________________________Page 4
- References to supplementary appendix ________________________________Page 5

ESM Figures

- ESM Fig.1____Page 6
- ESM Fig.2____Page 7

Human islets checklist____Page 8

ESM Methods

Blood samples collection and processing

Blood was drawn in the morning and all participants were instructed to fast for ≥10 hours before blood was drawn. Participants on insulin were also instructed to inject the last dose of basal insulin during the evening before the blood was drawn and not to inject short acting insulin during the five hours immediately preceding the blood drawn.

Blood samples were collected in K_3/K_2-EDTA Vacutainer tubes and processed using a standardized, two-step centrifugation protocol aimed at obtaining platelet-poor plasma with minimal cellular contamination. Briefly, after collection, the whole blood samples underwent an initial centrifugation for 10 minutes at 1800g at room temperature. Maintaining room temperature during this step is critical to avoid cold-induced platelet activation and subsequent release of contaminants (e.g. metabolites). The plasma layer was carefully transferred into a clean, RNase/DNase-free tube, posing attention not to disturb the buffy coat. This transferred plasma then underwent a second, lower-speed centrifugation for 20 minutes at 1200g at 10°C. This step is designed to remove any residual contaminant cells and cell debris, producing a highly purified plasma supernatant. Strict sterile techniques were enforced to prevent samples from contamination throughout the procedure.

Human Islets preparations and cytokine treatment

Human islet quality was assessed by static glucose-stimulated insulin secretion, and the insulin stimulation index (ISI) for each preparation was reported in the journal checklist. ISI values were as follows: Donor 1 (81 years), 6.3; Donor 2 (62 years), 3.5; Donor 3 (72 years), 4.9; Donor 4 (49 years), 3.9; Donor 5 (76 years), 1.9.

All donors died from cardiovascular disease, ensuring a homogeneous group of islet preparations and minimizing confounding effects related to alternative causes of death. Donors showed a mean glycemia of 119 ± 34 mg/dL during intensive care unit hospitalization (individual values reported in the checklist), indicating the absence of evident metabolic alterations.

Human islets were exposed to IL-1β (50 U/mL) and IFN-γ (1000 U/mL) for 48 hours. These cytokines, concentrations, and exposure time are widely validated and commonly used in the literature to

reproduce ex vivo the pro-inflammatory conditions of type 1 diabetes (T1D) islets. A 48-hour exposure induces islet dysfunction, activation of apoptotic pathways, and molecular alterations closely consistent with those observed in human T1D islets.

Dose–response analyses were not performed, as the selected cytokine concentrations are well established in the literature

Lipidomic analysis

Fasting serum lipids were extracted through protein precipitation. Specifically, 10 µL of blood serum, were mixed with 150 µL of cold methanol and 10 µL of internal standards (IS) mix including the following: Ceramide (CER (d18:1/17:0), 2.2 µM), Phosphatidylcholine (PC(17:0/17:0), 44.3 µM) and Lysophosphatidylcholine (LPC(17:0), 5.4 µM); phosphatidylethanolamine (PE(17:0/17:0), 34.3 µM), Sphingomyelin (SM(d18:1/17:0), 13.4 µM) (Avanti Polar Lipids, Alabaster, AL) and Triglycerides (TAG(15:0/15:0/15:0), 44.3 µM) (Larodan, Solna, SE). Samples were centrifuged (17,000 g x 20 minutes) and transferred into glass vials with insert and analyzed by LC/MS analysis.

In pancreatic islets (500,000 cells) lipids were extracted as described by Folch et al. [1]. Briefly, to each sample were added 0.9 mL of chloroform/methanol (2:1), the above-described lipid-mix of internal standards (10 µl), and for polar metabolites analysis 5 µl of a solution containing organic acid and amino acids mix standard labeled with stable isotopes at concentration 25 µM (Cambridge Isotope Laboratories, C.I.L., Cambridge, MA). Samples were then vortexed, centrifuged (17,000 g, 20 minutes) and 200 µL of milliQ water was added and samples were again vortexed and centrifuged (17,000 g, 10 minutes). The lower phase containing the lipid fraction was dried under a gentle steam of nitrogen and reconstituted with 10 µL of chloroform, vortexed and then added a further 150 µl of methanol.

Measurement of the lipidomic profile was performed by High-Performance Liquid Chromatography (Agilent UHPLC 1290 infinity) coupled with a Quadrupole Time-Of-Flight Mass Spectrometry QTOF (QTOF-MS, Agilent 6545) with electrospray ionization source (ESI), as previously reported [2]. For liquid chromatographic separation an Agilent ZORBAX Eclipse Plus C18 2.1 × 100 mm 1.8-Micron column was used and maintained at 50 °C. The mobile phase-A was water with 0.1% formic acid and the mobile phase-B was isopropanol/acetonitrile (1:1, v:v) with 0.1% formic acid. Injection volume was 1 µL.

The method allowed an untargeted acquisition of sample spectra in positive mode. Identification of lipid species was performed using a custom library containing more than 200 lipids species

belonging to seven different lipid classes, including phosphatidylcholines (PCs), phosphatidylethanolamines (PEs), lysophosphatidylcholines (LPCs), lysophosphatidylethanolamines (LPEs), triacylglycerols (TAGs), sphingomyelins (SMs) and ceramides (CERs), previously implicated in alterations of glucose and lipid metabolism and inflammation. Lipids were identified by retention time and m/z and spectral area was measured using Mass Hunter Profinder software (v.B.08.00, Agilent Technologies) and manually curated. Quantification of the lipids with spectral areas above the limit of detection (LOD) and a good signal-to-noise ratio was performed by calculating the ratio between the peak area corresponding to the target ion to the peak area of the internal standard [2]. Presence of outliers was assessed by principal component analyses.

Metabolomic analysis

For the analysis of metabolites, 60μL of serum were deproteinized with 300μL of cold methanol after adding labeled organic acid and amino acids mix standard (CIL Cambridge, MA, USA) and Folch's extraction procedure (volumetric ratio of 8:4:3 (v/v/v) chloroform/methanol/water) was used to extract the polar (aqueous) phase [1]. The upper phases of serum and pancreatic islets were dried under a gentle steam of nitrogen and polar metabolites were analyzed by High-Performance Liquid Chromatography (Agilent UHPLC 1290 infinity) coupled with Quadrupole Time-Of-Flight Mass Spectrometry QTOF (QTOF-MS, Agilent 6545) and by gas chromatography tandem mass spectrometry (Agilent Technology GC-8890/MS-7000 QQQ) using two 5-MS capillary columns in series (Agilent J&W GC Columns). For GC-MS analysis of amino acids and organic acids, extracted samples were derivatized in two-steps, i.e., first 30 μL of Methoxyamine 20 mg/mL (Merck, Germany) was added into test tube (30 min at 60 °C); then, after evaporating the residue to dryness under nitrogen at 40 °C, 30 μL of N-methyl-N-(tert-butyldimethylsilyl) trifluoroacetamide (MSTBSTFA, Merck, Germany). and 70 μL di Acetonitrile were added and incubated for 1h min at 60 °C [3, 4]. Amino acids and organic acids not detected in GC-MS analysis were measured by LC-MS QTOF analysis using an Agilent Infinity Lab Poroshell 120 HILIC-Z, 2.1 x 150 mm 2.7 μm column and Water Acquity UPLC BEH C18 2.1 x 100 mm 1.7 μm column for chromatographic separation. Negative ionization acquisition was performed using HILIC column was kept at 50 °C and chromatographic separation was achieved eluting the samples using a mixture of solvent A, 10 μM ammonium acetate in water, and solvent B, 10 μM ammonium acetate in water/acetonitrile (15:85, v:v), with a flow rate of 0.25mL/min and an injection volume of 3 μL. Positive ionization acquisition was performed using BEH C18 column kept at 50 °C and mobile phase-A was water with 0.1% formic

acid and the mobile phase-B was acetonitrile with 0.1% formic acid with a flow rate of 0.4mL/min and an injection volume of 3 µL.

Metabolite concentrations were quantified based on the ratio of the peak area of the target metabolite to the peak area of the corresponding labelled internal standard using MassHunter Profinder software (v.B.08.00, Agilent Technologies).

References

[1] Folch J, Lees M, Sloane Stanley GH (1957) A simple method for the isolation and purification of total lipides from animal tissues. J Biol Chem 226(1): 497-509

[2] Della Pepa G, Carli F, Sabatini S, et al. (2024) Clusters of adipose tissue dysfunction in adults with type 2 diabetes identify those with worse lipidomic profile despite similar glycaemic control. Diabetes Metab Res Rev 40(4): e3798. 10.1002/dmrr.3798

[3] Fenizia S, Scoditti E, Gastaldelli A (2023) Methods to Study Metabolomics. In: Federici M, Menghini R (eds) Gut Microbiome, Microbial Metabolites and Cardiometabolic Risk. Springer International Publishing, Cham, pp 1-41

[4] Gaggini M, Carli F, Rosso C, et al. (2018) Altered amino acid concentrations in NAFLD: Impact of obesity and insulin resistance. Hepatology 67(1): 145-158. 10.1002/hep.29465

Supplementary Figures

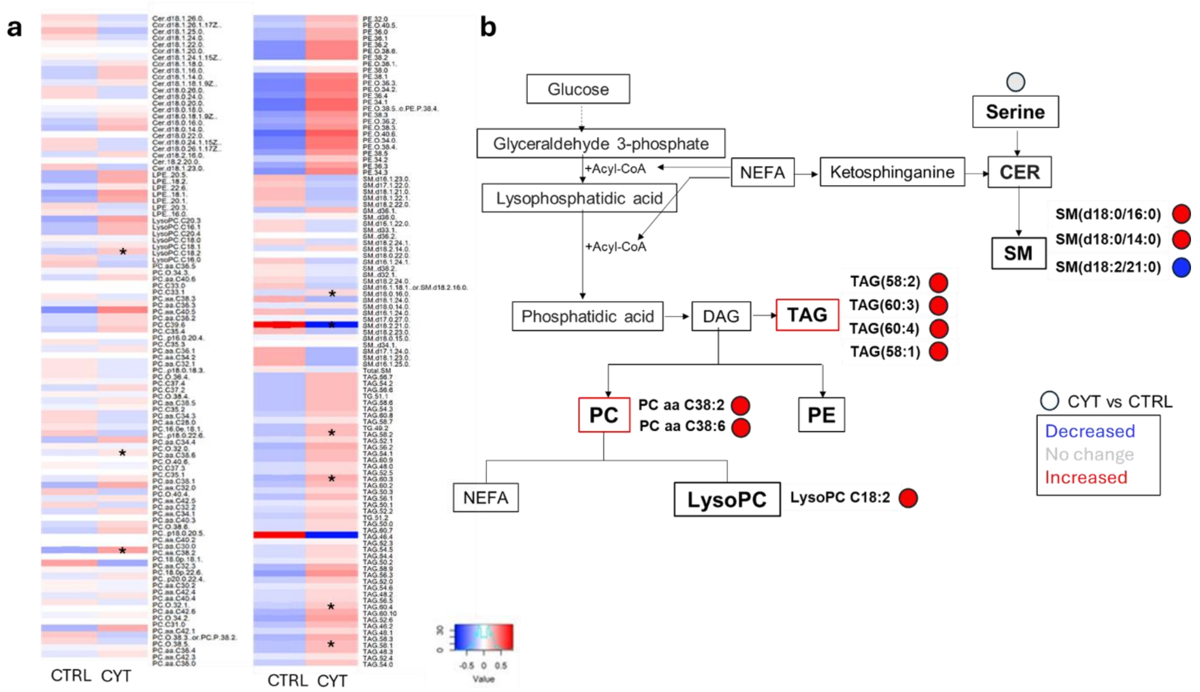

ESM Fig.1. **The concentration of lipids in pancreatic islets from healthy donors incubated with proinflammatory cytokines vs controls**. Panel a shows the heatmap of lipids in the 2 groups. Data were scaled to zero mean and unit variance and reported as mean within the two groups (group values are also presented in **ESM Table 10).** T-test p-value * vs controls <0.05, after adjusting for FDR. Panel b represents the pathways of lipids that are significantly different between the two groups. The color legend indicates relative concentrations: higher (red), lower (blue), no change (gray).

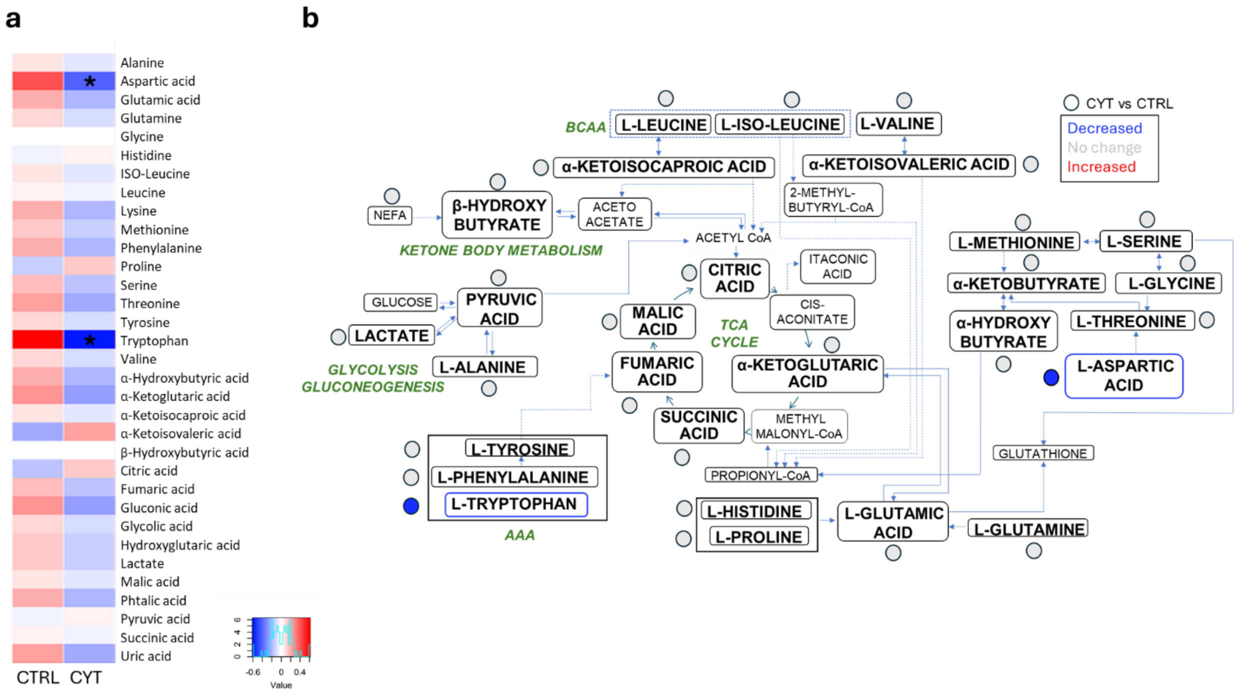

ESM Fig.2. **The concentration of polar metabolites in pancreatic islets from healthy donors incubated with proinflammatory cytokines vs controls**.

Panel a shows the heatmap of polar metabolites in the 2 groups. Data were scaled to zero mean and unit variance and reported as mean within the two groups (group values are also presented in **ESM Table 11).** T-test p-value * vs controls <0.05, after adjusting for FDR.

Panel b represents the polar metabolites of TCA cycle, glycolysis and some amino-acids pathways highlighting changes between the two groups. The color legend indicates relative concentrations: higher (red), lower (blue), no change (gray).

Checklist for reporting human islet preparations used in research

Adapted from Hart NJ, Powers AC (2018) Progress, challenges, and suggestions for using human islets to understand islet biology and human diabetes. Diabetologia https://doi.org/10.1007/s00125-018-4772-2

Islet preparation	1	2	3	4	5	6	7	8[a]
**MANDATORY INFORMATION**								
Unique identifier	27/20	27/31	27/48	27/114	27/120			
Donor age (years)	81	62	72	49	76			
Donor sex (M/F)	M	M	F	F	M			
Donor BMI (kg/m^2)	22.9	37.0	22.9	31.2	23.1			
Donor HbA_{1c} or other measure of blood glucose control (Mean glycemia during intensive unit care stay, mg/dl)	72	159	105	145	138			
Origin/source of islets[b]	Multiorgan donor	Multiorgan donor	Multiorgan donor	Multiorgan donor	Multiorgan donor			
Islet isolation centre	Pancreatic Islet Lab, University of Pisa	Pancreatic Islet Lab, University of Pisa	Pancreatic Islet Lab, University of Pisa	Pancreatic Islet Lab, University of Pisa	Pancreatic Islet Lab, University of Pisa			
Donor history of diabetes? Please select yes/no from drop down list	No	No	No	No	No			
**If Yes, complete the next two lines if this information is available**								
Diabetes duration (years)								
Glucose-lowering therapy at time of death[c]								
**RECOMMENDED INFORMATION**								
Donor cause of death	Cardiovascular disease	Cardiovascular disease	Cardiovascular disease	Cardiovascular disease	Cardiovascular disease			
Warm ischaemia time (h)	NA	NA	NA	NA	NA			
Cold ischaemia time (h)	14	17	13	12	13			
Estimated purity (%)	80	60	80	80	60			
Estimated viability (%)	NA	NA	NA	NA	NA			
Total culture time (h)[d]	96	96	96	96	96			
Glucose-stimulated insulin secretion or other functional measurement[e] (Static glucose-	6.3	3.5	4.9	3.9	1.9			

stimulated insulin secretion, values expressed as Insulin stimulation index)								
Handpicked to purity? Please select yes/no from drop down list	Yes	Yes	Yes	Yes	Yes			
Additional notes								

[a]If you have used more than eight islet preparations, please complete additional forms as necessary
[b]For example, IIDP, ECIT, Alberta IsletCore
[c]Please specify the therapy/therapies
[d]Time of islet culture at the isolation centre, during shipment and at the receiving laboratory
[e]Please specify the test and the results
